# Dissecting medial temporal lobe from diencephalic sub-volumes: The amnesia dichotomy revisited

**DOI:** 10.1162/IMAG.a.1205

**Published:** 2026-04-13

**Authors:** Célia Soussi, Léa Chauveau, Robin de Flores, Nicolas Cabé, Alice Laniepce, Laurent Coulbault, Céline Boudehent, Vincent de la Sayette, Gael Chételat, Shailendra Segobin, Anne-Lise Pitel

**Affiliations:** Normandie Univ, UNICAEN, INSERM, UA20, NEUROPRESAGE, Cyceron, Caen, France; Centre Hospitalier Universitaire de Caen, Caen, France; Normandie Univ, UNIROUEN, CRFDP (EA 7475), Rouen, France; Service de Biochimie, Centre Hospitalier Universitaire de Caen, Caen, France; Normandie Univ, UNICAEN, PSL Université, EPHE, INSERM, U1077, CHU de Caen, GIP Cyceron, NIMH, Caen, France; Institut Universitaire de France (IUF), Paris, France

**Keywords:** thalamic nuclei, mammillothalamic tract, medial temporal lobe, Korsakoff syndrome, alcohol use disorder, Alzheimer’s disease

## Abstract

Severe episodic memory deficits have historically been categorized as diencephalic amnesia, like in Korsakoff’s Syndrome (KS), or as medial temporal lobe (MTL) amnesia, like in Alzheimer’s Disease (AD). However, recent research has highlighted that both MTL and thalamus contribute to episodic memory. We aimed to challenge the traditional distinction by assessing whether subregional volume loss in the MTL and diencephalon reflects the distinct amnesia profiles traditionally associated with KS and AD. This cross-sectional observational study includes 203 subjects, comprising 81 healthy control participants. Of the 122 patients, 42 had amnesia: 18 patients with KS and 24 patients with AD at a dementia stage (dAD); and 80 had mild cognitive disorders: 50 patients with severe alcohol use disorder (AUD) without KS and 30 patients with amnestic mild cognitive impairment (aMCI). High-resolution T1-weighted MRI was used to quantify MTL subregions (anterior/posterior hippocampus, entorhinal cortex, parahippocampal cortex) using the ASHS-T1 pipeline and key diencephalic structures (anterior/mediodorsal thalamic nuclei, mammillothalamic tract) using the HIPS-THOMAS toolbox. Volume loss among patients and regions were compared using a linear mixed model. For each region, correlations between episodic memory and volumes loss were assessed and compared between aMCI/dAD and AUD/KS patients. Results showed no volume difference between patient groups for the anterior and posterior hippocampus and parahippocampal cortex. Entorhinal cortex was more altered in aMCI and dAD than in AUD and KS. KS and AUD patients showed disproportionate structural alterations in thalamic nuclei and mammillothalamic tracts compared with MTL subregions. KS patients showed more severe alterations than all other groups, except for anterior thalamic nuclei for which volume did not differ between KS and dAD. Episodic memory performance of AUD and KS patients correlated with volumes loss in the anterior and mediodorsal thalamic nuclei and mammillothalamic tract, while that of aMCI and dAD patients correlated with volume loss in the anterior hippocampus. Alterations in the MTL and diencephalon are not as clearly dissociated as traditionally proposed in the classification of amnesia. While KS pathology is characterized by more severe diencephalic alterations, only the entorhinal cortex is more damaged in AD than in KS. Neither hippocampal nor anterior thalamic volume loss appears to distinguish between the two types of amnesia, suggesting that structural changes in these two regions jointly participate to the pathophysiology of amnesia whatever the etiology. However, these alterations are not similarly involved in memory deficits, which suggests different architectural and/or functional implications of same-scale volume loss.

## 1 Introduction

The Papez circuit, also known as the memory circuit, was historically described as a loop originating in the hippocampus, connecting the anterior thalamus, mammillary bodies, cingulate cortex, and hippocampal formation (Papez, 1937). The latter includes the hippocampus proper (CA1–CA4) and the subiculum, both subcortical structures located within the medial temporal lobe (MTL). In line with a serial conception of the memory circuit, the MTL has been identified as a critical substrate for episodic memory. This assumption is supported by studies of amnesic patients, such as the famous HM case (Scoville & Milner, 1957), or investigations conducted in Alzheimer’s Disease (AD) (Braak & Braak, 1991b). AD is characterized by episodic memory disorders that worsen over time, eventually leading to amnesia at the dementia stage (dAD) that significantly impacts the patient’s autonomy. It is associated with pathological changes, such as hyperphosphorylated tau proteins leading to neurofibrillary tangles, originating in the MTL and beginning years before the onset of cognitive impairments (Braak & Braak, 1991b; Sun et al., 2024). To better understand the pathophysiological processes involved, studies have included patients with amnestic-type mild cognitive impairments (aMCI) and a biological profile of AD, making possible to capture prodromal changes associated with the first functional repercussion of the disease before the onset of dementia (Albert et al., 2011). Although aMCI patients have memory problems and can be classified as amnestic, in this paper we will refer to amnesia for conditions that severely impairs autonomy, and, therefore, applicable to dAD patients but not to aMCI patients.

A more recent work has established bidirectional interconnections within the Papez circuit, including projections from the thalamus to the hippocampal formation (Aggleton & Brown, 1999). This revision emphasizes the critical role of each structure within the network, notably the anterior thalamic nuclei (Aggleton & Brown, 1999), challenging the dominant view of the hippocampus as the exclusive center of memory (Aggleton, 2014; Aggleton et al., 2023). While the contribution of the diencephalon to episodic memory has long been recognized, it has received comparatively less sustained attention than hippocampal mechanisms. The contribution of the diencephalon to episodic memory has been evidenced through studies of patients with amnesia following stroke affecting the thalamus or mammillothalamic tract (Carlesimo et al., 2011; Danet et al., 2015), as well as in amnesic patients with Korsakoff’s Syndrome (KS) (Kopelman, 2022). KS is a persistent neurologic condition due to thiamine deficiency, mostly occurring in context of severe alcohol use disorder (AUD). To determine which brain alterations are specific to amnesia and not the consequences of alcohol-related damage, studies have compared KS patients to patients with AUD, since the latter can present alcohol-related episodic memory deficits but not amnesia (Harding et al., 2000; Pitel et al., 2012; Segobin et al., 2015, 2019). Amnesia in KS patients has been defined as diencephalic in agreement with significant alterations identified in mammillary bodies, anterior thalamic nuclei, and mammillothalamic tracts (Harding et al., 2000; Segobin et al., 2019; Yoneoka et al., 2021).

Historically, amnesia has thus been defined according to the location of the lesion identified as responsible for memory impairment (Scoville & Milner, 1957). In this framework, amnesia of the MTL, as in AD, has been opposed to diencephalic amnesia, as in KS (Kopelman, 2022). Yet, recent results have prompted us to revise these historical conceptions (Bernstein et al., 2021; De Simone et al., 2024; Forno et al., 2021). In fact, diencephalic alterations in AD are also of importance (Forno et al., 2021). Neuropathological studies showed that tau pathology and neurofibrillary tangles appear in the anterior thalamic nuclei at the same time as in the hippocampus (Braak & Braak, 1991b), with AD-related lesions confined mainly to the “limbic” thalamic nuclei (i.e., anterior and mediodorsal nuclei) while sparing the thalamus as a whole (Braak & Braak, 1991a; Rüb et al., 2016). This underlines the cruciality of considering the thalamus as a set of individual nuclei, yet in vivo segmentation of this structure is a methodological challenge (Segobin et al., 2024). The few neuroimaging studies that have examined volume loss in thalamic nuclei confirmed neuropathological data by establishing that both anterior and medial thalamic nuclei are altered in AD, in both aMCI and dAD patients (Bernstein et al., 2021; De Simone et al., 2024). One study found that episodic memory was associated with volume loss in anterior and mediodorsal nuclei in AD patients and healthy controls pooled together (Bernstein et al., 2021) while another one, which only investigated correlations in AD patients, found no significant link between episodic memory and volumes loss in those nuclei (De Simone et al., 2024). Also, in both AUD and KS patients, the hippocampus and other regions of the MTL are altered (Sullivan & Marsh, 2003; Visser et al., 1999; Wilson et al., 2017) (see also Squire et al. (1990)). Hippocampal volume appears related to memory impairments in KS patients (Sullivan & Marsh, 2003) (see also Visser et al. (1999)), while no link was demonstrated between hippocampal volume and episodic memory performance in AUD patients (Chanraud et al., 2009; Fama et al., 2021). This could suggest a particular implication of the hippocampus in KS only.

Very few studies have directly compared the MTL or diencephalic volumes of patients with amnesia due to AD or to KS (Segobin et al., 2023; Sullivan & Marsh, 2003), and fewer have used segmentation techniques to compare subregions or nuclei volumes (Segobin et al., 2023). Results have highlighted that hippocampal alterations are of the same extent in dAD patients and KS patients (Segobin et al., 2023; Sullivan & Marsh, 2003), even though hippocampal alterations were more severe in dAD patients at an advanced stage of the disease than in KS patients (Segobin et al., 2023). The thalamus as a whole appeared more affected in KS patients (Segobin et al., 2023). Interestingly, when comparing specific nuclei of the thalamus, authors have found that the anterior thalamic nuclei were altered to the same extent, while the medial thalamic nuclei were more affected in KS (Segobin et al., 2023). This highlights the importance of considering the thalamus as an assembly of distinct nuclei.

To be able to challenge the traditional diencephalic *versus* MTL amnesia classification, direct comparisons are lacking, and three main gaps can be identified in the existing literature. First, both the MTL and thalamus must be considered as a collection of subregions/nuclei that should be examined separately. Recent reliable methods are now available for accurately and automatically segmenting the two structures in subregions from *in vivo* images (Vidal et al., 2024; Yushkevich et al., 2015). This level of precision has never been reported in *in vivo* comparisons of KS and dAD patients. Secondly, inclusion of patients with milder episodic memory deficits that do not affect their autonomy (i.e., AUD and aMCI) may help to discern which brain alterations emerge early and which alterations are specifically associated with amnesia. This approach provides a clearer picture of the trajectory leading to amnesia, enabling us to distinguish general markers of disease progression from alterations that are truly characteristic of KS and dAD patients. Third, the lack of clear links between sub-volumes changes and episodic impairments limits our understanding of their functional relevance. Segmentation of subregions *in vivo* would allow us to link specific structural alteration and memory impairments.

Our study aimed to address these gaps by comparing volume loss in MTL and diencephalic subregions between patients with KS and dAD using state-of-the-art segmentation methods. To better understand the structural alterations underlying each type of amnesia, we also included patients with mild neurocognitive disorders in the comparisons (AUD and aMCI). Specifically, our study is part of the process of challenging the classification of diencephalic *versus* MTL amnesia by (1) comparing volume loss in subregions of both structures and (2) exploring the relationships between regional volume loss and episodic memory deficits.

## 2 Methods

### 2.1 Protocol and participants

In this comparative, retrospective cross-sectional study, we used baseline data from two observational and longitudinal studies conducted in the same neuroimaging center (Cyceron, Caen, France) during the same period. Studies were conducted on the same MRI camera using the same imaging protocol, and shared a part of the neuropsychological evaluation. The IMAP Study (registered with http://clinicaltrials.gov, number NCT01638949) aimed at investigating the relevance of different brain measures in the prediction of AD progression of cognitively healthy adults (HC) and patients at different stages of AD (inclusion and data collection between January 2008 and October 2016). ALCOBRAIN/ALCOSLEEP study (registered with http://clinicaltrials.gov, number NCT01455207) aimed at screening for cognitive impairments and brain damage in AUD and KS patients (inclusion and data collection between October 2012 and July 2019). All participants were at least 18 years old and were native French speakers. Participants had no history of cerebrovascular disease, psychiatric disorders, head trauma, drug abuse (except tobacco for all participants and alcohol for AUD and KS patients), neurologic disease (other than KS or AD for the corresponding groups), or other major diseases such as cancer. No participant was under psychotropic medication with the exception of four KS patients who had been on stable medication for several years, with no recent changes. Both the IMAP and ALCOBRAIN/ALCOSLEEP studies were approved by the Comité de Protection des Personnes Nord-Ouest III in France. All participants (or their caregivers when necessary) provided their written informed consent for inclusion in either protocol. These studies were carried out in line with the Declaration of Helsinki (1964).

HC participants had to be cognitively unimpaired and to have performance in the normal range in all neuropsychological tests. aMCI and dAD patients were recruited in local memory clinics and met DSM-5 criteria for mild or major AD-related neurocognitive disorders as well as internationally agreed criteria for probable AD (McKhann et al., 1984; Petersen & Morris, 2005). Patients with severe form of dAD were not included to avoid difficulties in carrying out the protocol. AUD patients were recruited as inpatients in the addiction department of Caen University Hospital at the end of their withdrawal period (i.e., patients had been free of benzodiazepine withdrawal treatment and symptoms for at least 48 hours, corresponding to a mean of 11 ± 4.67 days of abstinence (min = 8; max = 24)) and met the DSM-IV criteria for alcohol dependence (American Psychiatric Association, 1994) or DSM-5 criteria for severe AUD (American Psychiatric Association, 2013). KS patients were either recruited as inpatients or in a nursing home, and were diagnosed with KS as defined by the DSM-5 criteria for alcohol-induced major neurocognitive disorders, amnestic-confabulatory type, persistent (American Psychiatric Association, 2013) or by the DSM-IV criteria for amnesia due to substance abuse (American Psychiatric Association, 1994). Part of the latter diagnosis involved patients showing amnesia on repeated neuropsychological assessments. All patients were diagnosed by experienced clinicians after careful investigation. Our different groups have already been included in other studies (e.g., for AUD and KS groups (Morand et al., 2024; Ritz et al., 2022; Segobin et al., 2023) and for aMCI, dAD, and HC groups (Chauveau et al., 2021; Kuhn et al., 2023)).

We only included participants who had performed an MRI examination with satisfactory quality control (e.g., no severe motion artifacts). Amyloid status of IMAP participants was assessed based on their 18F-AV45 PET (fluorine-18 florbetapir positron emission tomography) cortical SUVr (standardized uptake value ratio) transformed to Centiloids (Klunk et al., 2015; Navitsky et al., 2018) (See Supplementary Method for details). We included AD patients (aMCI and dAD) with a positive amyloid status and HC with a negative amyloid status, using a cutoff of 12 Centiloid (La Joie et al., 2019).

Even if we are using baseline data only, the longitudinal clinical follow-up conducted in IMAP allowed to reassess the AD diagnosis up to 15 years after inclusion. A diagnostic committee composed of a multidisciplinary team of experts reassessed AD diagnosis, when possible, based on information from the longitudinal study and consultation with clinicians involved in patient care after study completion. Clinical information about AUD and KS patients was also consulted in September 2024 (5 to 12 years after inclusion) to assess whether any neurodegenerative disease was suspected at a distance from inclusion. One KS patient later received a diagnosis of AD and was excluded from this study.

We included 50 patients with AUD (age = 46.89 ± 8.90 years, 88% men), 30 with aMCI (age = 73.76 ± 6.64 years, 66% men), 18 with KS (age = 53.83 ± 4.80 years, 44% men), and 24 with dAD (age = 67.78 ± 9.89 years, 66% men), as well as 80 HC participants (age = 47.30 ± 19.80 years, 55% men). Frequency of male was higher in AUD patients than in KS and HC groups. aMCI and dAD patients were significantly older than HC participants and AUD and KS patients. Patients were significantly less educated than HC participants. All patients were impaired on the Mini-Mental State Examination (MMSE) when compared with HC participants, with KS and dAD patients having significantly lower performance than aMCI and AUD patients, coherently with the definition of groups in terms of symptoms severity. Episodic memory was assessed with the delayed free recall of the Free and Cued Selective Reminding Test (/16) (FCSRT, French version), (Van der Linden et al., 2004). For a few patients (*N = *4), only two recalls of the FCSRT out of three during the learning phase could be administered due to their difficulties. Even though the differed recall was systematically conducted, we chose not to include those patients in analyses involving this score because of different administration procedures. A gradient in episodic memory performance was observed, with KS and dAD patients having the lowest scores compared with HC participants, then MCI patients, and finally AUD patients. Group characteristics are described in Table 1.

**Table 1.** Demographics and cognitive scores.

	AUD	aMCI	KS	dAD	HC	Statistics	Post Hoc comparisons
Demographics							
*N*	50	30	18	24	81		
Sex/male *N* (%)	44 (88%)	20 (66%)	8 (44%)	16 (66%)	45 (55%)	X² = 18.31 *p* *=* 0.001 *V* = 0.289	AUD ≠ (MCI = KS = MA = HC)
Age *years*	46.89 (8.90) [26-66]	73.76 (6.64) [60-85]	53.83 (4.80) [44-62]	67.78 (9.89) [54-84]	47.30 (19.8) [20-81]	*F*(4,198)=28.37 *p < *0.001 *η*2 = 0.364	(HC = AUD = KS) < (aMCI = dAD)
Education *years*	11.86 (2.10) [9-17]	11.66 (4.17) [6-20]	10.06 (2.26) [6-15]	11.17 (3.40) [6-20]	13.48 (3.19) [7-20]	*F*(4,198)=6.70 *p < *0.001 *η2* = 0.119	(AUD = aMCI = KS = dAD) < HC
Global cognition							
*N*	27	30	18	23	81		
MMSE *(max=30)*	27.18 (2.70) [20–30]	26.83 (1.82) [22–30]	22.61 (3.79) [12–27]	20.74 (4.93) [12–29]	29.10 (0.99) [26–30]	*F*(4,174)=60.60 *p < *0.001 *η2* = 0.584	(K S = dAD) < (aMCI = AUD) < HC
Episodic memory							
*N*	49	27	17	17	46		
Delayed free recall (FCSRT) *(max=16)*	10.63 (3.05) [0–14]	4.93 (3.00) [0–10]	2.88 (3.08) [0–11]	2.88 (2.32) [0–8]	12.91 (2.11) [9–16]	*F(*4,149)=73.67 *p < *0.001 *η2* = 0.694	KS = dAD < aMCI < AUD < HC

Data are N (%) or mean (sd) [range].

AUD: patients with alcohol use disorder; aMCI: patients with amnestic-type mild cognitive impairment; KS: patients with Korsakoff syndrome; dAD: patients with Alzheimer’s disease; MMSE: Mini-Mental State Examination; FCSRT: Free and Cued Selective Reminding Test; NA: not applicable.

Groups were compared with χ² or ANOVAs. Post hoc test: pairwise comparisons or Tukey’s HSD. Effect sizes are specified with Cramer’s V or *η2.*

### 2.2 Segmentation of subregions

#### 2.2.1 MRI T1 acquisition

High-resolution T1-weighted anatomical images were obtained on a Philips Achieva 3T scanner with a 3D fast-field echo sequence with the following parameters: 180 sagittal slices; thickness = 1 mm, repetition time (TR) = 20 ms, echo time (TE) = 4.6 ms, flip angle = 10°, field of view (FOV) = 256 x 256 mm^2^. T1 images were segmented into gray matter, white matter, and cerebrospinal fluid using SPM12. The total intracranial volume (TIV) was calculated based on the sum of the three individual volumes.

#### 2.2.2 Segmentation of the medial temporal lobe (MTL)

**Fig. 1.**
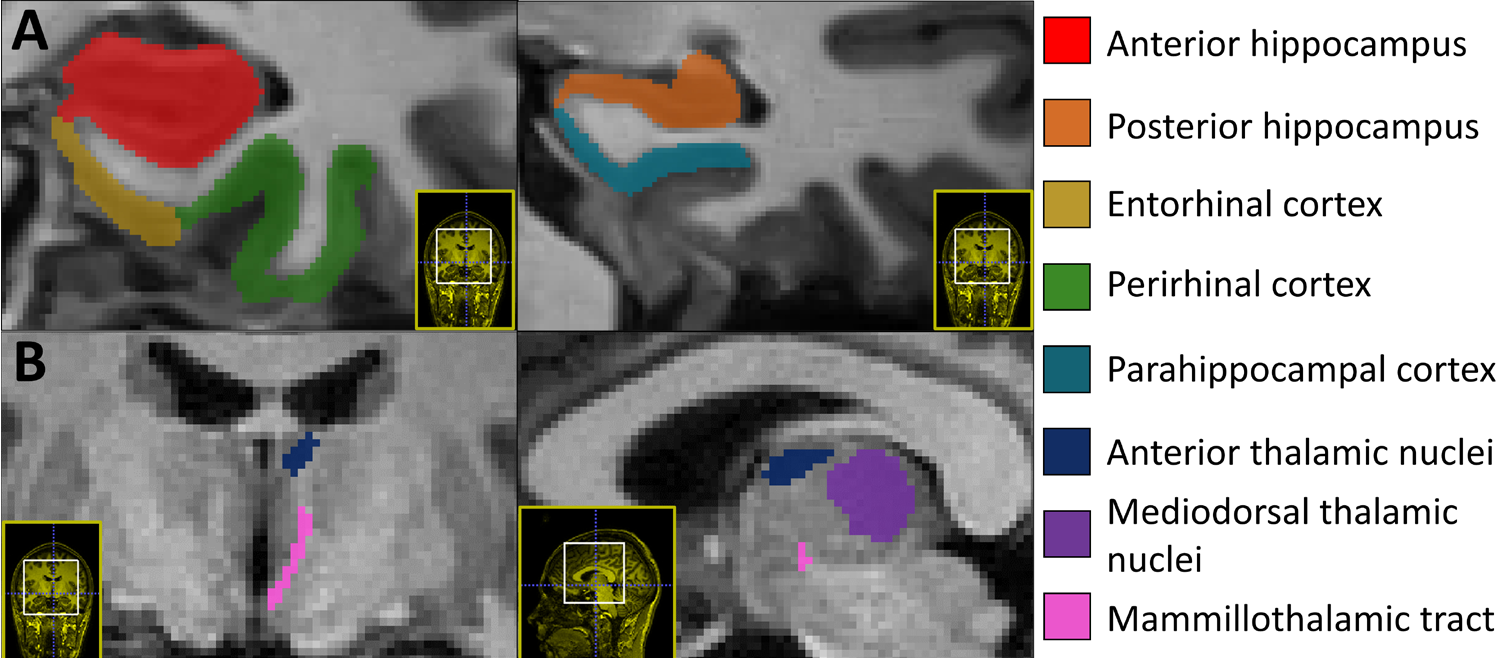
Example of unilateral segmentation of the medial temporal lobe. (A) Medial temporal lobe segmentation obtained with the ASHS-T1 pipeline, shown on two coronal slices. (B) Diencephalic segmentation obtained with the HIPS-THOMAS algorithm is rendered in one coronal (*left*) and one sagittal (*right*) slice.

For each individual’s T1w-MR images, unilateral MTL was segmented into anterior hippocampus, posterior hippocampus, entorhinal cortex, perirhinal cortex (Broadman areas 35 and 36), and parahippocampal cortex with the ASHS-T1 pipeline (Fig. 1A) (Yushkevich et al., 2015). All segmented results went through rigorous quality check prior to quantitative analysis. Manual correction was applied to failed segmentation when feasible, for the hippocampus only (70 unilateral segmentations, 8.62% of hippocampal segmentations), using ITK-SNAP software (Yushkevich et al., 2006). When impossible to manually correct, unilateral failed segmentations were discarded (see Table 2 for details). Quality control and manual corrections were performed by two experimenters. To ensure consistency, both independently processed a subsample of participants (*N* = 10). For each subregion, less than 3% of all unilateral segmentations failed and was not manually corrected, except for the perirhinal cortex for which it was frequent (26%). This can be explained both by anatomical specificities and high interindividual variability in this subregion.

**Table 2.** Mean volume loss by group and by region.

		AUD	KS	aMCI	dAD
Medial Temporal Lobe	*N* *				
Anterior hippocampus	401	-0.37 (0.85) [-2.43; 2.21]	-0.55 (0.78) [-2.39; 1.18]	-0.91 (0.99) [-3.45; 0.87]	-0.99 (0.64) [-2.31; 0.14]
Posterior hippocampus	396	-0.48 (1.03) [-2.77; 2.04]	-0.31 (1.23) [-2.43; 2.28]	-1.30 (1.24) [-3.99; 1.83]	-1.26 (0.93) [-3.61; 0.82]
Entorhinal cortex	403	-0.11 (1.02) [-2.64; 2.91]	-0.30 (1.09) [-2.92; 1.67]	-1.14 (1.08) [-3.69; 1.27]	-1.47 (1.06) [-3.97; 1.03]
Perirhinal cortex	298	-0.38 (0.92) [-2.30; 1.73]	-0.68 (0.99) [-2.95; 1.39]	-1.06 (0.94) [-3.57; 1.26]	-1.10 (1.06) [-3.25; 1.52]
Parahippocampal cortex	401	-0.77 (0.99) [-3.16; 1.48]	-0.58 (0.81) [-2.14; 1.62]	-0.72 (0.89) [-2.62; 1.64]	-0.78 (0.79) [-2.32; 1.26]
Diencephalon	*N* *				
Anterior thalamic nuclei	406	-0.69 (0.86) [-2.76; 1.16]	-1.64 (0.74) [-3.22; 0.50]	-1.24 (0.65) [-3.01; -0.05]	-1.29 (0.66) [-2.62; -0.02]
Mediodorsal thalamic nuclei	406	-0.99 (0.86) [-3.35; 1.13]	-2.23 (0.84) [-3.49; -0.44]	-1.17 (0.75) [-3.14; 0.42]	-1.34 (0.95) [-4.25; 0.20]
Mammillothalamic tract	406	-0.82 (1.14) [-3.32; 2.02]	-2.39 (1.15) [-4.72; -0.17]	-0.96 (0.86) [-2.83; 0.98]	-0.94 (0.82) [-3.70; 0.92]

Volume loss is expressed in z-scores (mean of 0 and standard deviation of 1 in the control group). Means are specified for each group and each region (sd) [range].

AUD = patients with alcohol use disorder; KS = patients with Korsakoff’s Syndrome; aMCI = patients with amnestic-type mild cognitive impairment; dAD = patients with Alzheimer’s Disease at a dementia stage.

* Number of unilateral volumes per subregions. Unilateral segmentation failed and impossible to manually correct for 5 anterior hippocampus, 10 posterior 3 entorhinal cortex, 108 perirhinal cortex and 5 parahippocampal cortex.

#### 2.2.3 Segmentation of the thalamus

For each individual’s T1w-MR images, unilateral thalamus was segmented using HIPS-THOMAS algorithm (Vidal et al., 2024). This pipeline allows segmentation into 12 nuclei and the mammillothalamic tract based on the Morel nomenclature (Morel et al., 1997). We selected the anterior thalamic nuclei (“anteroventral nuclei”), mediodorsal thalamic nuclei (“mediodorsal-parafascicular nucleus”), and mammillothalamic tract based on their relevance to episodic memory (Fig. 1B). Thalamic segmentation is currently an acknowledged methodological challenge, which notably limits the definition of other anterior nuclei *in vivo* (Segobin et al., 2024). A study comparing the efficiency of available state-of-the-art methods of thalamic segmentation has demonstrated that HIPS-THOMAS was overall the best method for segmentation of anterior and mediodorsal thalamic nuclei as well as the best method to discriminate AD from HC and capture AD evolution (Williams et al., 2024). We must underline that this method does not allow the isolation of the mediodorsal nuclei from the parvocellular and magnocellular nuclei. Thalamic segmentations also went through rigorous quality control. Manual corrections were carried out in ITK-SNAP software (Yushkevich et al., 2006), for a sub-sample of unilateral volumes in the pulvinar region where the segmentation did not perform to satisfaction (14 unilateral segmentation, 2.29% of total diencephalic segmentations).

#### 2.2.4 Computation of a volume loss value

Each unilateral volume extracted from the segmentations was normalized by the TIV [normalized volume = raw volume / (TIV*1000)]. Normalized volumes were standardized using a z-score transformation based on the HC group. By definition, these z-scores had a mean of 0 and a standard deviation of 1 for the HC group. A negative value would reflect volume loss compared with the mean volume of the HC group. These transformations allowed measures reflecting volume loss in a comparable way between each patient and each region, independently of the size of the region. These values were used for group comparisons.

To limit number of tests in analyses when investigating links with cognition, individual bilateral volume loss was computed for each region as the means of left and right values. If one value was missing, bilateral volume was not computed.

### 2.3 Statistical analyses

#### 2.3.1 Comparisons of volumes

Statistical analyses were carried out using R Statistical Software (v4.2.3). To compare volume loss in the four groups of patients, we ran a linear mixed effect model controlling for age, sex, and region laterality, incorporating fixed effects for the interaction between groups and regions and a random participant effect using the *“lme4”* R package (Bates et al., 2015). Age was modeled using a quadratic (age+age^2^) fit because quadratic models systematically provided a better fit than linear models for explaining age-related variations in volume across all subregions and groups, consistent with previous findings (Lee et al., 2022). The “*compare_performance*” R package (Lüdecke et al., 2021) was used to select the best model and assess its quality. The selected model had the best compromise between explanatory performance (high marginal R²) and parsimony (low Akaike Information Criterion (AIC) and Bayesian Information Criterion (BIC)), see Supplementary Table S2 for details. The quality assessment indicated that the selected model was not singular and did not exhibit heteroscedasticity, as confirmed by the *check_heteroskedasticity()* test from the “*performance*” package (*p* = 0.838), supporting the assumption of homoscedastic residual variance.

Pairwise comparisons were computed on estimated marginal means adjusted for covariates using the “*emmeans*” R package to investigate between- and within-group differences. For between-group differences, comparisons were adjusted with a Tukey HSD method. For within-group comparisons, planned contrasts were designed to compare subregions between the two different structures only (i.e., comparing each MTL subregion to each thalamic nuclei, but comparisons within MTL subregions or within thalamic nuclei were not performed). Planned comparisons, conducted via custom contrasts, were additionally adjusted for multiple comparisons using the Holm method.

Significant results are reported at a 5% alpha level (*p* < 0.05).

#### 2.3.2 Links between volumes loss and memory scores

For all regions and all groups, volumes were normally distributed. However, the episodic memory score (i.e., delayed free recall of the FCSRT) was not. Spearman partial correlations controlling for age were calculated for AUD and KS patients pooled together and for aMCI and dAD patients pooled together to compare relationships between bilateral regional volume losses and episodic memory scores in each etiology. Pooling increased intra-group variability and statistical sensitivity, while preserving meaningful comparisons between distinct etiological profiles. On an exploratory basis, correlation coefficients within each group are reported for all regions without multiple comparison correction, using a significance threshold of *p* < 0.05.

For each region, correlation coefficients were then compared with two-tailed Fisher’s *Z* tests using the R package *cocor* (Diedenhofen & Musch, 2015) between AUD and KS patients on the one hand and dAD and aMCI patients on the other hand. Given the multiple comparisons, p-values from these tests were adjusted using the Holm method to control the family-wise error rate. Adjusted p-values (*p*_corr_) are reported and considered significant at *p* < 0.05.

## 3 Results

### 3.1 Volume loss comparisons

Descriptive data of volume loss for each region and for each group are available in Table 2 (see Supplementary Table S1 for descriptive data of raw volumes expressed in mm^3^).

The linear mixed model revealed a significant interaction between group and region (*F*(18,1541.630) = 15.182, *p < *0.001), warranting post hoc comparisons.

#### 3.1.1.1 Volume loss in the medial temporal lobe (MTL)

Regarding the entorhinal cortex, dAD patients had more severe volume loss than AUD patients (*t = *5.200, *p < *0.001, 95% CI [0.592; 1.762]) and KS patients (*t = *3.707, *p = *0.001, 95% CI [0.284; 1.586]). aMCI patients also had more severe volume loss than AUD patients (*t = *3.209, *p = *0.008, 95% CI [0.150; 1.403]).

No significant group differences in volume loss were observed in the others region.

Regarding the posterior hippocampus, the AUD group exhibited less severe volume loss than dAD patients (*t = *2.534, *p = *0.057, 95% CI [-0.012; 1.153]) and the KS group exhibited less severe volume loss compared with dAD patients (*t = *2.539; *p = *0.056, 95% CI [-0.011; 1.306]) but the differences did not reach significance.

#### 3.1.1.2 Volume loss in the thalamus and mammillothalamic tracts

For the anterior thalamic nuclei, KS patients had more severe volume loss than AUD patients (*t = *4.490, *p < *0.001, 95% CI [0.415; 1.538]) and aMCI patients (*t = *2.790, *p = *0.029, 95% CI [0.053; 1.395]). Volume loss did not differ significantly between KS and dAD patients despite a tendency toward more severe volume loss in KS patients (*t = *-2.342, *p = *0.091, 95% CI [-1.241; 0.060]).

For the mediodorsal thalamic nuclei and mammillothalamic tracts, KS patients had more severe volumes loss than all other groups (*p < *0.001) who did not differ between each other.

#### 3.1.2 Between-region differences within each group

**Fig. 2.**
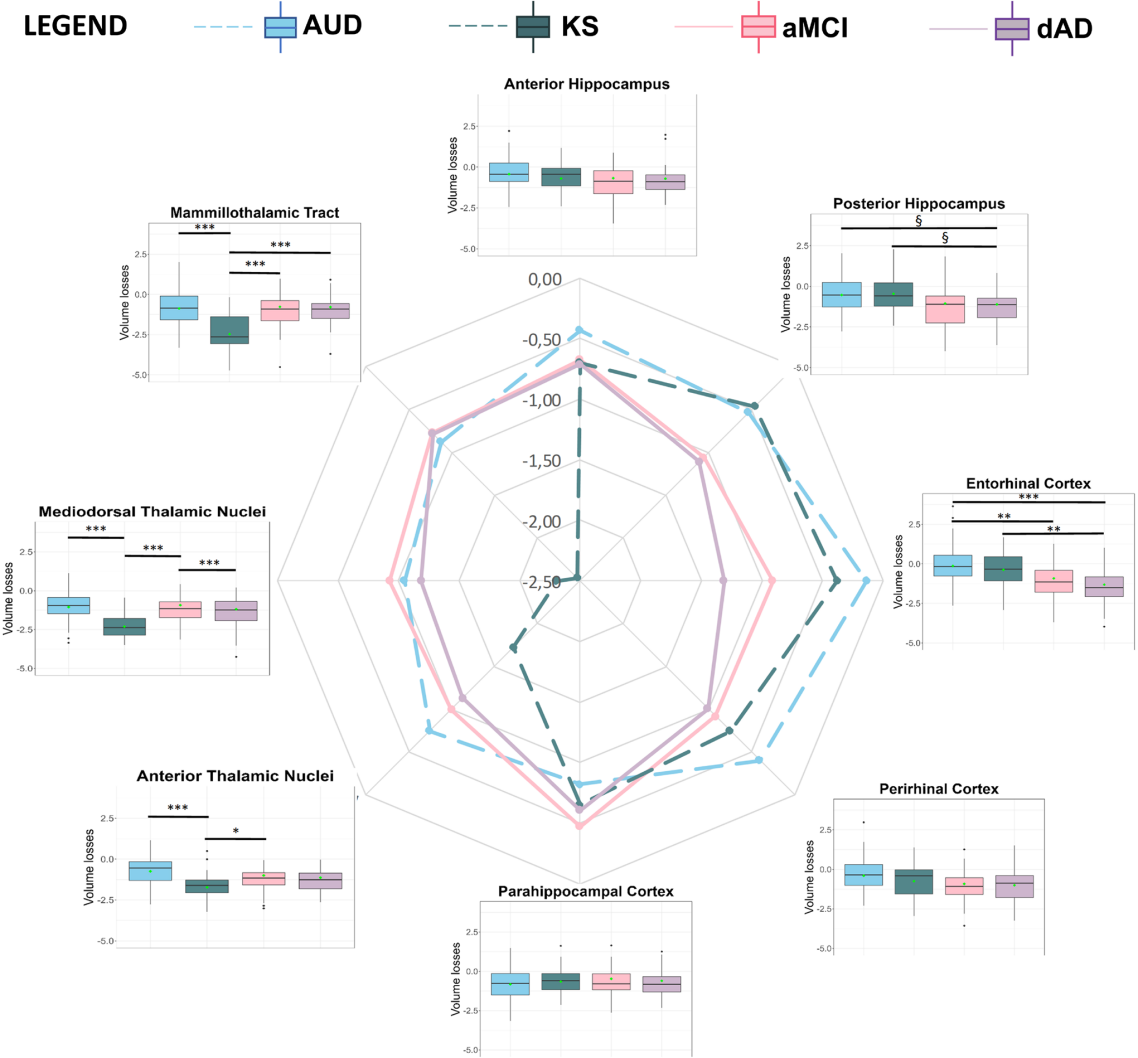
Representation of volume loss by group and by region. For each region and each patient group, the boxplot displays volume loss distributions. Estimated marginal means are shown with a green dot on the boxplots and displayed on a radar chart. Statistical differences from pairwise comparisons are indicated. Between-group differences are flagged: *** *p < *0.001; ** *p < *0.01; * *p < *0.05; § *p < *0.06. AUD = patients with alcohol use disorder; KS= patients with Korsakoff’s Syndrome; aMCI = patients with amnestic-type mild cognitive impairment; dAD = patients with Alzheimer’s Disease at a dementia stage.

Pairwise comparisons of volumes loss between regions were run within each group. Statistically significant differences could not be flagged in Figure 2 but are illustrated in Supplementary Figure S1.

##### 3.1.2.1 Patients with AUD and KS

For AUD patients, volume loss in the mediodorsal thalamic nuclei and of the mammillothalamic tracts was more severe than those in the anterior hippocampus (separately: *t = *-5.385, *p < *0.001, 95% CI [-0.952; -0.280]; *t = *-3.884, *p < *0.001, 95% CI [-0.780; -0.108]), posterior hippocampus (separately: *t = *-4.498, *p < *0.001, 95% CI [-0.850; -0.178]; *t = *-2.997, *p = *0.019, 95% CI [-0.679; -0.007]), entorhinal cortex (separately: *t = *-7.987, *p < *0.001, 95% CI [-1.249; -0.577]; *t = *-6.487, *p < *0.0001, 95% CI [-1.078; -0.406]), and perirhinal cortex (separately: *t *= -5.564*, p* < 0.001, 95% CI [-0.986; -0.304]; *t *= -4.083, *p* < 0.001, 95% CI [-0.814; -0.133]). Volume loss in the anterior thalamic nuclei was more severe than that in the entorhinal cortex (*t = *-5.337, *p < *0.001, 95% CI [-0.946; -0.274]) and anterior hippocampus (*t *= -2.734, *p = *0.032, 95% CI [-0.649; 0.023]).

For KS patients, volumes loss in the anterior and mediodorsal thalamic nuclei, as well as in the mammillothalamic tracts, was more severe than all MTL subregions (*p < *0.001).

##### 3.1.2.2 Patients with aMCI and AD

For both aMCI and dAD patients, volume loss in the anterior and mediodorsal thalamic was more severe than that of the parahippocampal cortex (aMCI: *t = *-3.547, *p* = 0.006, 95% CI [-0.957; -0.090] and *t = *-3.064, *p* = 0.031, 95% CI [-0.886; -0.018], respectively; dAD: *t = *-3.116, *p* = 0.024, 95% CI [-1.017; -0.030] and *t = *-3.448, *p = *0.009, 95% CI [-1.073; -0.086], respectively).

For dAD patients only, volume loss in the mediodorsal thalamic nuclei was more severe than that of the anterior hippocampus (*t = *-2.880, *p* = 0.048, 95% CI [-1.073; -0.086]), and volume loss in the entorhinal cortex was more severe than that of the mammillothalamic tracts (*t* *=* 3.202, *p* = 0.019, 95% CI [0.043; 1.1013]).

### 3.2 Links between volume loss and episodic memory

**Fig. 3.**
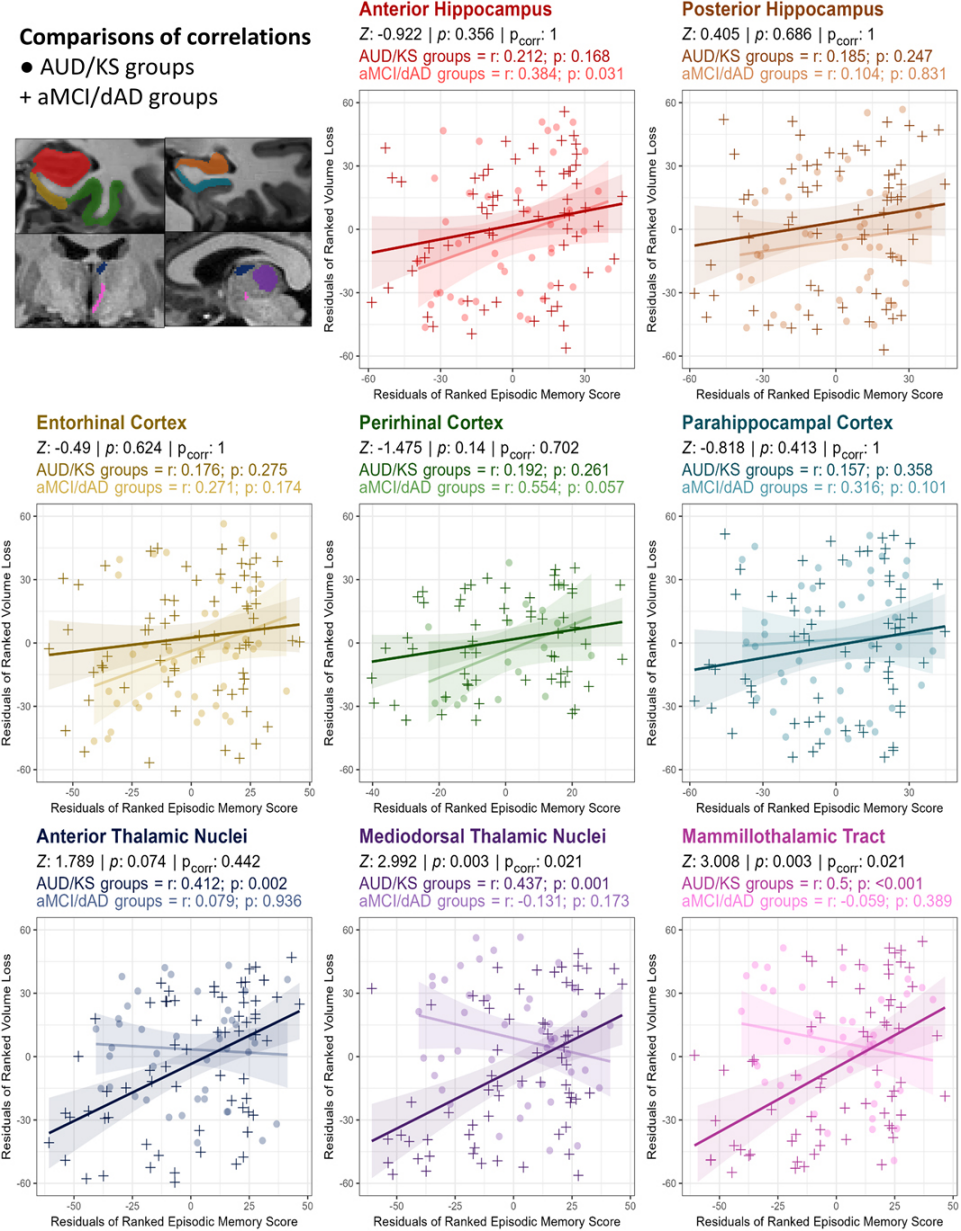
Partial correlations (controlled by age) between regional volume loss and episodic memory performance in AUD and KS patients pooled together and aMCI and dAD patients pooled together. For each region, scatterplots illustrate the partial correlations controlling for age (age + age^2^) between episodic memory score (Delayed Free Recall of the FCSRT) and volume loss for AUD + KS patients and aMCI + dAD patients. Regional volume loss is color coded according to the corresponding anatomical region shown in the *top-left* panel. Within each plot, the AUD/KS group is depicted with darker-colored points shaped as crosses, while the aMCI/AD group is shown with lighter-shaded round points for visual comparison. Spearman’s correlation coefficients (*rho*) and their p-values are displayed for each group. Correlation coefficients were compared between patients with alcohol-related disorders (AUD + KS) and AD patients (aMCI + AD) using Fisher’s Z test, *Z* values, p-values, and p-values corrected for multiple comparisons with the Holm method (*p*_corr_) are shown on each panel. *Note:* plots show residuals from age-adjusted regressions on ranked data for illustration, while statistics are partial Spearman correlations controlling for age.

In AUD and KS patients combined, lower episodic memory performance was associated with higher volume loss in the two investigated thalamic nuclei (anterior thalamic nuclei: *rho* = 0.41, *p* *=* 0.002; mediodorsal thalamic nuclei: *rho* = 0.44, *p* *=* 0.001) as well as in the mammillothalamic tracts (*rho* = 0.50, *p* < 0.001). No significant correlations were found between episodic memory results and MTL volume loss (Fig. 3).

In aMCI and dAD patients combined, lower episodic memory performance was selectively associated with more severe volume loss in the anterior hippocampus (*r* = 0.38, *p* = 0.031).

Comparisons of correlations (Fig. 3) between AUD and KS patients on one hand and aMCI and dAD patients on the other hand showed significant differences for the mediodorsal thalamic nuclei (*Z* = 2.99, *p*_corr_ *=* 0.021) and mammillothalamic tract (*Z* = 3.01, *p*_corr_ *=* 0.021).

## 4 Discussion

Using state-of-the-art methods of segmentation, our goal was to reassess the relevance of the diencephalic *versus* MTL amnesia classification, not only by investigating volume loss at the subregion level, but also its functional relevance to memory. To do so, we (1) compared volume loss in subregions of both structures, depending on etiology and in attempt to determine brain abnormalities specific to amnesia and (2) investigated the links between structural alterations and episodic memory for each etiology. We provide both anatomical and functional arguments that partly support, but also significantly challenge, the diencephalic *versus* MTL amnesia classification.

### 4.1 Evidence in favor of the traditional classification between MTL and diencephalic amnesia

#### 4.1.1 MTL damage in amnesia due to AD

Our refined anatomical segmentation highlights that only a subregion of the MTL, the entorhinal cortex, is more significantly affected in aMCI and dAD patients compared with AUD and KS patients. The originality of the present study is also to directly compare the extent of MTL and diencephalic volume loss. While no structural differences between MTL and diencephalon is observed in aMCI patients, in dAD only the entorhinal cortex is more severely altered than the mammillothalamic tracts. Taken together, these results unveil a preferential alteration of the MTL in AD confined to the entorhinal cortex. This aligns with the pathway of tau pathology in AD and the associated atrophy, which typically begins in the entorhinal cortex before spreading to the hippocampus and neocortical regions (Braak & Braak, 1991b). The entorhinal is among the first subregion to degenerate, thus, it is expected to be more severely impaired. There is no clear predominance of MTL damage in aMCI and dAD patients, which will be discussed further below in the light of recent findings on early thalamic vulnerability in AD pathology.

#### 4.1.2 Diencephalic damage in amnesia due to KS

In all investigated diencephalic regions, KS patients display more severe alterations than AUD or aMCI patients. Regarding the anterior and mediodorsal thalami and the mammillothalamic tract, alterations are more severe in KS patients than in AUD patients. KS patients also have disproportionate thalamic and mammillothalamic alterations compared with MTL alterations. In AUD patients, this distinction is less clear, notably with no significant difference between diencephalic subregions and the parahippocampal cortex. This further underlines the importance of the thalamus and mammillothalamic tract in KS severe episodic memory impairments and supports the definition of this amnesia as diencephalic (Harding et al., 2000; Yoneoka et al., 2021). In this perspective, it has been proposed that disconnection within the mammillothalamic tract (Segobin et al., 2015) or the fornix (Segobin et al., 2019) would provoke alterations in the anterior thalamic nuclei that would be responsible of amnesia in KS.

#### 4.1.3 Structure–function dissociations across etiologies

Investigation of the links between structural alterations and verbal episodic memory unveils a dissociation between structure and memory function depending on the etiology. In AUD and KS patients, verbal memory performance was only linked to thalamic and mammillothalamic tract volumes loss, while in aMCI and AD patients, episodic memory deficits were specifically related to volume loss in the anterior hippocampus. These findings are consistent with neuropathological and neuroimaging studies describing that most amnesic KS patients displayed the most severe diencephalic volume loss (Shimamura et al., 1988; Squire et al., 1990). There is also robust evidence that volume of the anterior hippocampus negatively correlates to episodic memory in AD (Chételat et al., 2003; Leube et al., 2008). It appears that amnesia in KS only relates to diencephalic structural alteration, while amnesia in AD only relates to hippocampal alterations, supporting the functional dichotomization of these two etiologies into diencephalic *versus* MTL amnesia. However, this implication can be challenged.

### 4.2 Challenges to the dichotomic classification

#### 4.2.1 Diencephalic damage in amnesia due to AD

Despite AD being labeled as MTL amnesia and previously described results, a few thalamic nuclei are affected as much or even more than certain MTL regions. In aMCI patients, the diencephalon was affected to the same extent as the anterior and posterior hippocampus, and alterations in both anterior and mediodorsal thalamic nuclei are even more severe than in the parahippocampal cortex. Same observations were made in dAD patients, with more severe alterations in the mediodorsal thalamic nuclei than in the anterior hippocampus. This is consistent with early thalamic alterations in AD pathology (Braak & Braak, 1991b), occurring even before alterations to a few MTL regions, and aligns with a shift in AD literature from an MTL-centered concept to models encompassing other subcortical and cortical regions, and notably the thalamus (Aggleton et al., 2016; Argyropoulos et al., 2019; Forno et al., 2021). Coherently, we also underline that while volume loss is more severe in the mammillothalamic tracts and the mediodorsal nuclei in KS patients than in dAD patients, similar alterations are found in the anterior thalamic nuclei, replicating results from a previous study including the same KS patients but different patients with AD (Segobin et al., 2023). This is consistent with the early vulnerability of the anterior thalamic nuclei in AD pathology (Braak & Braak, 1991a, 1991b), as well as its role as a hub in the Papez Circuit (Aggleton et al., 2022). This nucleus is reciprocally connected to the hippocampal formation with which it jointly contributes to episodic memory (Aggleton et al., 2022, 2023; Segobin et al., 2019; Vann & Albasser, 2009). Previous studies about diencephalic amnesia beyond KS have underlined that structural damage in the diencephalon is associated with hippocampal hypoactivity, modeling the importance of inputs from the anterior thalamic nuclei to hippocampus (Vann & Albasser, 2009). We can hypothesize that similar mechanisms are involved in AD, with alteration of the anterior thalamic nuclei majoring hippocampal alterations and vice versa. Thus, early alterations of the anterior thalamic nuclei in AD could be both directly and indirectly responsible for progressive deterioration of memory, and ultimately amnesia. In this context, our findings further encourage the growing interest in the role of this subregion in AD (Bernstein et al., 2021; De Simone et al., 2024; Forno et al., 2023).

#### 4.2.2 MTL damage in amnesia due to KS

Regarding the hippocampus, anterior and posterior alterations do not statistically differ, neither between aMCI and AUD patients, nor between dAD and KS patients. This is consistent with previous comparisons of the two latter groups of patients (Segobin et al., 2023; Sullivan & Marsh, 2003), though hippocampal alterations were more severe in dAD patients with more advanced diseases (Segobin et al., 2023). This could suggest that AD is not more of a “hippocampal amnesia” than KS, and that hippocampal alterations could rather be a common feature of memory disorders. Still, studies of amnesic patients have demonstrated that very localized lesions in other nodes of the memory circuit (Carlesimo et al., 2011; Danet et al., 2015) can provoke amnesia without any structural damage in the hippocampus. Thus, rather than a localizationist concept of amnesia, considering the latter as the result of network-level alterations would better explain repercussions of damage in one structure to the other nodes of the network. In this context, a tripartite model of diencephalic–hippocampal interactions (Aggleton & O’Mara, 2022) offers a valuable framework. It distinguishes three core circuits involving the hippocampus, anterior thalamic nuclei, and associated cortical structures, each contributing to the episodic memory system. According to this view, damage to one node (e.g., anterior thalamic nuclei) can disrupt the entire memory system, leading to amnesia even without direct hippocampal damage. Similar hippocampal alterations in all patients’ groups lead us to suggest that secondary damage to the hippocampus, either functional, microstructural, and/or structural, may be a systematically encountered feature in amnesic patients, while direct hippocampal lesions are not necessary for amnesia, as shown by the current lack of memory–hippocampal associations in patients with AUD and KS.

Still, we must qualify our conclusions by underlining that in the present study, dAD patients tend to have more severe volume loss in the posterior hippocampus than KS patients (*p* = 0.057). Future studies should compare hippocampal subfields between dAD and KS patients. Previous research suggests that CA2 + 3 could be especially vulnerable to AUD, whereas CA1 is consistently reported as an early vulnerable region in AD (de Flores et al., 2015; Zahr et al., 2019). Such investigation in KS could show a different pattern of impairments and we hypothesize that subfields more pre-eminently present in the anterior hippocampus are more severely altered.

#### 4.2.3 Structure–function dissociations across etiologies

We have previously stated that there is an apparent structure–function dissociation across etiologies that is congruent with the classical MTL *versus* diencephalic amnesia. However, this conclusion must be qualified, as we shall describe below.

Despite being an apparent structural hallmark of AD, we did not find any significant correlations between episodic memory performance and the volume of the entorhinal cortex in AD patients, nor with the posterior hippocampus. This contrasts with consistent literature linking episodic memory and volume of the entorhinal cortex (Chauveau et al., 2021; Di Paola et al., 2007) and of the posterior hippocampus (Chauveau et al., 2021) in AD. This discrepancy could be explained by our unique measure of episodic memory that prevents us from investigating modalities other than verbal memory. Investigating both visual and spatial memory could be more sensitive to capture links with entorhinal and hippocampal damage (Chauveau et al., 2021), notably the posterior hippocampus that is more specialized in navigation and spatial processing (Grady, 2020), and more involved in tasks of visual memory, such as face recognition (Poppenk et al., 2013). The use of a multimodal measure of episodic memory could also help establish links between memory and thalamic structural alterations in AD patients. In addition, we only used a measure of differed recall, which lacks procedural specificity. For example, we could expect the anterior hippocampus to be more involved in encoding processes and the posterior hippocampus to be more involved in retrieval processes (Grady, 2020). The use of an experimental task allowing to isolate encoding, retrieval, and storage would be relevant in future studies.

Moreover, even if macrostructural alterations are not linked to functional impairment, the latter may relate to microstructural (Chanraud et al., 2009; De Simone et al., 2024) or functional alterations (Morand et al., 2024). Herein, comparisons of correlations for the anterior hippocampus between AUD/KS patients and aMCI/dAD patients were not significant. This could suggest that hippocampal involvement in memory may exist in AUD and KS patients, without being detected in our sample. Links between hippocampal volume and memory performance have already been observed in a small sample of KS patients (Sullivan & Marsh, 2003), but was not observed in a more extended sample (Visser et al., 1999), nor in AUD patients (Chanraud et al., 2009; Fama et al., 2021). Still, less commonly used imaging methods have revealed that episodic memory relates to microstructural integrity in the hippocampus of AUD patients (Chanraud et al., 2009), and that of the anterior thalamus in aMCI or AD patients (De Simone et al., 2024).

Despite these considerations, present results linking structural alterations and verbal memory remain important to consider, notably because this is the first evidence of different functional relevance of subregions depending on etiologies involved.

### 4.3 Reconciling the two perspectives: toward a functional dissociation?

Our results show similar structural damage with volume loss in both the hippocampus and the anterior thalamic nuclei in amnesia due to AD or KS. Same-scale alterations in these two key nodes of the Papez circuit are coherent with the tripartite model proposing that conjoint damage to both structure leads up to amnesia (Aggleton et al., 2016, 2023; Aggleton & O’Mara, 2022). However, despite similar structural alterations, the relationships between volume loss in the anterior hippocampus and episodic memory were not observed in AUD and KS patients, and relationships between volume loss in the anterior thalamic nuclei were absent in aMCI and dAD patients. This highlights a functional dissociation since structural alterations with comparable severity relate differently to functional impairments. This result aligns with the idea that memory dysfunction cannot be attributed solely to lesions in isolated regions but rather to network-level alterations (Aggleton et al., 2016, 2023; Argyropoulos et al., 2019; La Joie et al., 2014). The cingulate cortex is thought of as the leading candidate among the cortical areas of memory consolidation that integrate information from both the hippocampus and anterior thalamic nuclei (Aggleton & O’Mara, 2022). In that context, studying both the integrity of the cingulate and its connectivity with the hippocampus and anterior thalamic nuclei would be an important perspective for our work. The same anatomical changes may have different functional consequences depending on the broader abnormalities in the circuit, which differ according to the nature of amnesia. Thus, such findings highlight the limitations of a dichotomous view of amnesia.

### 4.4 Limitations

Our study compared patients from rare, well-selected and diverse populations allowing nuanced comparisons across etiologies and disease stages. The integrity of our regions of interest was assessed with subregional anatomical precision using state-of-the-art methods of segmentation. By combining volumetric analyses with episodic memory correlations, the study links regional brain alterations to cognitive outcomes, strengthening the neurobiological interpretation of findings. However, it presents certain limits.

First, its cross-sectional approach hampers the inferences that can be drawn about the potential evolution from AUD/aMCI to amnesia in KS/dAD. It is also important to highlight that while aMCI patients can be considered as in a prodromal stage of dAD, it is not the case for AUD and KS patients. Still, both dAD and KS patients were, respectively, aMCI and AUD patients before the onset of amnesia. Second, although all analyses were corrected for age, the groups were not age matched. Characteristics of each pathology hamper the ability to recruit patients in the same age range, but future studies should replicate present results in age-matched groups. Even though excessive alcohol consumption was an exclusion criterion for patients with aMCI and dAD, no precise quantification of alcohol consumption was conducted in these groups. Similarly, no AD biomarker was available for patients with AUD and KS, even though the long-term clinical follow-up provided additional reassurance by allowing the retrospective exclusion of suspected cases of neurodegenerative diseases. Other limitations of the study, particularly with regard to examining the relationship between brain volume and memory, include the modest sample sizes and the fact that only one verbal measure of episodic memory was available for all patient groups. Future studies could benefit from the integration of a multidimensional assessment of episodic memory. Finally, images of higher resolution could improve the precision of segmentation, enabling the study of hippocampal subfields, and multimodal imaging could be relevant to investigate network-level alterations.

### 4.5 Conclusions

Our results highlight the fact that alterations in the MTL and diencephalon are not as clearly dissociated as is traditionally proposed in the classification of amnesia. Interestingly, both the hippocampus and the anterior thalamic nuclei are similarly affected in KS and dAD, as well as in AUD and aMCI. This is consistent with models suggesting that both subregions jointly contribute, within an extended circuit, to memory function (Aggleton et al., 2022, 2023; Aggleton & Brown, 1999). However, similar structural alterations are differently linked to memory alterations depending on the etiology, adding a layer of complexity to our understanding of amnesia. Future studies should investigate other measures of cerebral integrity and their links to both verbal and visual episodic memory, to better understand the contribution of each impaired node within the memory circuit in different types of amnesia. Overall, our results favor moving away from a rigid neuroanatomical dichotomy of amnesia and instead suggest a functional network-based model, where different etiologies preferentially affect certain subregions and similarly affect others, but with distinct functional implications. While existing theories and animal models have emphasized the joint contribution of the medial temporal and diencephalic regions to episodic memory, this study provides one of the first clinical illustration to support these models, based on a wide spectrum of pathologies in human.

## Supplementary Material

Supplementary Material

## Data Availability

The data used in this study are available from the corresponding author upon reasonable request.

## References

[IMAG.a.1205-b1] Aggleton, J. P. (2014). Looking beyond the hippocampus: Old and new neurological targets for understanding memory disorders. Proceedings of the Royal Society B: Biological Sciences, 281(1786), 20140565. 10.1098/rspb.2014.0565 PMC4046414 24850926

[IMAG.a.1205-b2] Aggleton, J. P., & Brown, M. W. (1999). Episodic memory, amnesia, and the hippocampal-anterior thalamic axis. The Behavioral and Brain Sciences, 22(3), 425–444; discussion 444–489. 10.1017/s0140525x99002034 11301518

[IMAG.a.1205-b3] Aggleton, J. P., Nelson, A. J. D., & O’Mara, S. M. (2022). Time to retire the serial Papez circuit: Implications for space, memory, and attention. Neuroscience & Biobehavioral Reviews, 140, 104813. 10.1016/j.neubiorev.2022.104813 35940310 PMC10804970

[IMAG.a.1205-b4] Aggleton, J. P., & O’Mara, S. M. (2022). The anterior thalamic nuclei: Core components of a tripartite episodic memory system. Nature Reviews Neuroscience, 23(8), 505–516. 10.1038/s41583-022-00591-8 35478245

[IMAG.a.1205-b5] Aggleton, J. P., Pralus, A., Nelson, A. J. D., & Hornberger, M. (2016). Thalamic pathology and memory loss in early Alzheimer’s disease: Moving the focus from the medial temporal lobe to Papez circuit. Brain, 139(7), 1877–1890. 10.1093/brain/aww083 27190025 PMC4939698

[IMAG.a.1205-b6] Aggleton, J. P., Vann, S. D., & O’Mara, S. M. (2023). Converging diencephalic and hippocampal supports for episodic memory. Neuropsychologia, 191, 108728. 10.1016/j.neuropsychologia.2023.108728 37939875

[IMAG.a.1205-b7] Albert, M. S., DeKosky, S. T., Dickson, D., Dubois, B., Feldman, H. H., Fox, N. C., Gamst, A., Holtzman, D. M., Jagust, W. J., Petersen, R. C., Snyder, P. J., Carrillo, M. C., Thies, B., & Phelps, C. H. (2011). The diagnosis of mild cognitive impairment due to Alzheimer’s disease: Recommendations from the National Institute on Aging-Alzheimer’s Association workgroups on diagnostic guidelines for Alzheimer’s disease. Alzheimer’s & Dementia, 7(3), 270–279. 10.1016/j.jalz.2011.03.008 PMC3312027 21514249

[IMAG.a.1205-b8] American Psychiatric Association. (1994). Diagnostic and statistical manual of mental disorders (4th ed.). APA. https://psycnet.apa.org/record/1994-97698-000

[IMAG.a.1205-b9] American Psychiatric Association. (2013). Diagnostic and statistical manual of mental disorders (5th ed.). APA. https://dsm.psychiatryonline.org/doi/book/10.1176/appi.books.9780890425596

[IMAG.a.1205-b10] Argyropoulos, G. P. D., Loane, C., Roca-Fernández, A., Lage-Martínez, C., Gurau, O., Irani, S., & Butler, C. (2019). Network-wide abnormalities explain memory variability in hippocampal amnesia. eLife, 8, e46156. 10.7554/eLife.46156 31282861 PMC6639076

[IMAG.a.1205-b11] Bates, D., Mächler, M., Bolker, B., & Walker, S. (2015). Fitting linear mixed-effects models using lme4. Journal of Statistical Software, 67(1), 1–48. 10.18637/jss.v067.i01

[IMAG.a.1205-b12] Bernstein, A. S., Rapcsak, S. Z., Hornberger, M., Saranathan, M., & Alzheimer’s Disease Neuroimaging Initiative. (2021). Structural changes in thalamic nuclei across prodromal and clinical Alzheimer’s disease. Journal of Alzheimer’s Disease: JAD, 82(1), 361–371. 10.3233/JAD-201583 34024824

[IMAG.a.1205-b13] Braak, H., & Braak, E. (1991a). Alzheimer’s disease affects limbic nuclei of the thalamus. Acta Neuropathologica, 81(3), 261–268. 10.1007/BF00305867 1711755

[IMAG.a.1205-b14] Braak, H., & Braak, E. (1991b). Neuropathological stageing of Alzheimer-related changes. Acta Neuropathologica, 82(4), 239–259. 10.1007/BF00308809 1759558

[IMAG.a.1205-b15] Carlesimo, G. A., Lombardi, M. G., & Caltagirone, C. (2011). Vascular thalamic amnesia: A reappraisal. Neuropsychologia, 49(5), 777–789. 10.1016/j.neuropsychologia.2011.01.026 21255590

[IMAG.a.1205-b16] Chanraud, S., Leroy, C., Martelli, C., Kostogianni, N., Delain, F., Aubin, H.-J., Reynaud, M., & Martinot, J.-L. (2009). Episodic memory in detoxified alcoholics: Contribution of grey matter microstructure alteration. PLoS One, 4(8), e6786. 10.1371/journal.pone.0006786 19707568 PMC2728538

[IMAG.a.1205-b17] Chauveau, L., Kuhn, E., Palix, C., Felisatti, F., Ourry, V., de La Sayette, V., Chételat, G., & de Flores, R. (2021). Medial temporal lobe subregional atrophy in aging and Alzheimer’s disease: A longitudinal study. Frontiers in Aging Neuroscience, 13, 750154. https://www.frontiersin.org/articles/10.3389/fnagi.2021.750154 34720998 10.3389/fnagi.2021.750154 PMC8554299

[IMAG.a.1205-b18] Chételat, G., Desgranges, B., de la Sayette, V., Viader, F., Berkouk, K., Landeau, B., Lalevée, C., Le Doze, F., Dupuy, B., Hannequin, D., Baron, J., & Eustache, F. (2003). Dissociating atrophy and hypometabolism impact on episodic memory in mild cognitive impairment. Brain, 126(9), 1955–1967. 10.1093/brain/awg196 12821520

[IMAG.a.1205-b19] Danet, L., Barbeau, E. J., Eustache, P., Planton, M., Raposo, N., Sibon, I., Albucher, J.-F., Bonneville, F., Peran, P., & Pariente, J. (2015). Thalamic amnesia after infarct: The role of the mammillothalamic tract and mediodorsal nucleus. Neurology, 85(24), 2107–2115. 10.1212/wnl.0000000000002226 26567269 PMC4691690

[IMAG.a.1205-b20] de Flores, R., La Joie, R., & Chételat, G. (2015). Structural imaging of hippocampal subfields in healthy aging and Alzheimer’s disease. Neuroscience, 309, 29–50. 10.1016/j.neuroscience.2015.08.033 26306871

[IMAG.a.1205-b21] De Simone, M. S., Spalletta, G., Vecchio, D., Bassi, A., Carlesimo, G. A., & Piras, F. (2024). The role of the anterior thalamic nuclei in the genesis of memory disorders in Alzheimer’s disease: An exploratory study. Journal of Alzheimer’s Disease, 97(1), 507–519. 10.3233/JAD-230606 38189755

[IMAG.a.1205-b22] Di Paola, M., Macaluso, E., Carlesimo, G. A., Tomaiuolo, F., Worsley, K. J., Fadda, L., & Caltagirone, C. (2007). Episodic memory impairment in patients with Alzheimer’s disease is correlated with entorhinal cortex atrophy. Journal of Neurology, 254(6), 774–781. 10.1007/s00415-006-0435-1 17404777

[IMAG.a.1205-b23] Diedenhofen, B., & Musch, J. (2015). cocor: A comprehensive solution for the statistical comparison of correlations. PLoS One, 10(4), e0121945. 10.1371/journal.pone.0121945 25835001 PMC4383486

[IMAG.a.1205-b24] Fama, R., Le Berre, A.-P., Sassoon, S. A., Zahr, N. M., Pohl, K. M., Pfefferbaum, A., & Sullivan, E. V. (2021). Memory impairment in alcohol use disorder is associated with regional frontal brain volumes. Drug and Alcohol Dependence, 228, 109058. 10.1016/j.drugalcdep.2021.109058 34610518 PMC8595873

[IMAG.a.1205-b25] Forno, G., Lladó, A., & Hornberger, M. (2021). Going round in circles—The Papez circuit in Alzheimer’s disease. European Journal of Neuroscience, 54(10), 7668–7687. 10.1111/ejn.15494 34656073

[IMAG.a.1205-b26] Forno, G., Saranathan, M., Contador, J., Guillen, N., Falgàs, N., Tort-Merino, A., Balasa, M., Sanchez-Valle, R., Hornberger, M., & Lladó, A. (2023). Thalamic nuclei changes in early and late onset Alzheimer’s disease. Current Research in Neurobiology, 4, 100084. 10.1016/j.crneur.2023.100084 37397807 PMC10313877

[IMAG.a.1205-b27] Grady, C. L. (2020). Meta-analytic and functional connectivity evidence from functional magnetic resonance imaging for an anterior to posterior gradient of function along the hippocampal axis. Hippocampus, 30(5), 456–471. 10.1002/hipo.23164 31589003

[IMAG.a.1205-b28] Harding, A., Halliday, G., Caine, D., & Kril, J. (2000). Degeneration of anterior thalamic nuclei differentiates alcoholics with amnesia. Brain, 123(1), 141–154. 10.1093/brain/123.1.141 10611128

[IMAG.a.1205-b29] Klunk, W. E., Koeppe, R. A., Price, J. C., Benzinger, T. L., Devous, M. D., Jagust, W. J., Johnson, K. A., Mathis, C. A., Minhas, D., Pontecorvo, M. J., Rowe, C. C., Skovronsky, D. M., & Mintun, M. A. (2015). The Centiloid Project: Standardizing quantitative amyloid plaque estimation by PET. Alzheimer’s & Dementia: The Journal of the Alzheimer’s Association, 11(1), 1–15.e1–4. 10.1016/j.jalz.2014.07.003 PMC4300247 25443857

[IMAG.a.1205-b30] Kopelman, M. D. (2022). What is the Korsakoff syndrome?—A paper in tribute to Prof Alwyn Lishman. Cognitive Neuropsychiatry, 27(4), 296–313. 10.1080/13546805.2022.2067472 35477346

[IMAG.a.1205-b31] Kuhn, E., Perrotin, A., La Joie, R., Touron, E., Dautricourt, S., Vanhoutte, M., Vivien, D., de La Sayette, V., Chételat, G., & for the Alzheimer’s Disease Neuroimaging Initiative. (2023). Association of the informant-reported memory decline with cognitive and brain deterioration through the Alzheimer Clinical Continuum. Neurology, 100(24), e2454–e2465. 10.1212/WNL.0000000000207338 37085328 PMC10264050

[IMAG.a.1205-b32] La Joie, R., Ayakta, N., Seeley, W. W., Borys, E., Boxer, A. L., DeCarli, C., Doré, V., Grinberg, L. T., Huang, E., Hwang, J.-H., Ikonomovic, M. D., Jack, C., Jagust, W. J., Jin, L.-W., Klunk, W. E., Kofler, J., Lesman-Segev, O. H., Lockhart, S. N., Lowe, V. J.,… Rabinovici, G. D. (2019). Multisite study of the relationships between *antemortem* [11C]PIB-PET Centiloid values and *postmortem* measures of Alzheimer’s disease neuropathology. Alzheimer’s & Dementia, 15(2), 205–216. 10.1016/j.jalz.2018.09.001 PMC6368897 30347188

[IMAG.a.1205-b33] La Joie, R., Landeau, B., Perrotin, A., Bejanin, A., Egret, S., Pélerin, A., Mézenge, F., Belliard, S., de La Sayette, V., Eustache, F., Desgranges, B., & Chételat, G. (2014). Intrinsic connectivity identifies the hippocampus as a main crossroad between Alzheimer’s and semantic dementia-targeted networks. Neuron, 81(6), 1417–1428. 10.1016/j.neuron.2014.01.026 24656258

[IMAG.a.1205-b65] Lee, H., Chen, C., Kochunov, P., Elliot Hong, L., & Chen, S. (2022). Modeling multivariate age-related imaging variables with dependencies. Statistics in Medicine, 41(22), 4484–4500. 10.1002/sim.9522 36106648 PMC9494615

[IMAG.a.1205-b34] Leube, D. T., Weis, S., Freymann, K., Erb, M., Jessen, F., Heun, R., Grodd, W., & Kircher, T. T. (2008). Neural correlates of verbal episodic memory in patients with MCI and Alzheimer’s disease—A VBM study. International Journal of Geriatric Psychiatry, 23(11), 1114–1118. 10.1002/gps.2036 18449954

[IMAG.a.1205-b35] Lüdecke, D., Ben-Shachar, M. S., Patil, I., Waggoner, P., & Makowski, D. (2021). Performance: An R Package for assessment, comparison and testing of statistical models. Journal of Open Source Software, 6(60), 3139. 10.21105/joss.03139

[IMAG.a.1205-b36] McKhann, G., Drachman, D., Folstein, M., Katzman, R., Price, D., & Stadlan, E. M. (1984). Clinical diagnosis of Alzheimer’s disease: Report of the NINCDS‐ADRDA Work Group* under the auspices of Department of Health and Human Services Task Force on Alzheimer’s disease. Neurology, 34(7), 939–939. 10.1212/WNL.34.7.939 6610841

[IMAG.a.1205-b37] Morand, A., Laniepce, A., Cabé, N., Boudehent, C., Segobin, S., & Pitel, A.-L. (2024). Compensation patterns and altered functional connectivity in alcohol use disorder with and without Korsakoff’s syndrome. Brain Communications, 6(5), fcae294. 10.1093/braincomms/fcae294 39309684 PMC11414044

[IMAG.a.1205-b38] Morel, A., Magnin, M., & Jeanmonod, D. (1997). Multiarchitectonic and stereotactic atlas of the human thalamus. Journal of Comparative Neurology, 387(4), 588–630. 10.1002/(SICI)1096-9861(19971103)387:4<588::AID-CNE8>3.0.CO;2-Z 9373015

[IMAG.a.1205-b39] Navitsky, M., Joshi, A. D., Kennedy, I., Klunk, W. E., Rowe, C. C., Wong, D. F., Pontecorvo, M. J., Mintun, M. A., & Devous Sr., M. D. (2018). Standardization of amyloid quantitation with florbetapir standardized uptake value ratios to the Centiloid scale. Alzheimer’s & Dementia, 14(12), 1565–1571. 10.1016/j.jalz.2018.06.1353 30006100

[IMAG.a.1205-b40] Papez, J. W. (1937). A proposed mechanism of emotion. Archives of Neurology & Psychiatry, 38(4), 725–743. 10.1001/archneurpsyc.1937.02260220069003

[IMAG.a.1205-b41] Petersen, R. C., & Morris, J. C. (2005). Mild cognitive impairment as a clinical entity and treatment target. Archives of Neurology, 62(7), 1160–1163. 10.1001/archneur.62.7.1160 16009779

[IMAG.a.1205-b42] Pitel, A.-L., Chételat, G., Le Berre, A. P., Desgranges, B., Eustache, F., & Beaunieux, H. (2012). Macrostructural abnormalities in Korsakoff syndrome compared with uncomplicated alcoholism. Neurology, 78(17), 1330–1333. 10.1212/WNL.0b013e318251834e 22496200

[IMAG.a.1205-b43] Poppenk, J., Evensmoen, H. R., Moscovitch, M., & Nadel, L. (2013). Long-axis specialization of the human hippocampus. Trends in Cognitive Sciences, 17(5), 230–240. 10.1016/j.tics.2013.03.005 23597720

[IMAG.a.1205-b44] Ritz, L., Segobin, S., Laniepce, A., Lannuzel, C., Boudehent, C., Vabret, F., Urso, L., Pitel, A. L., & Beaunieux, H. (2022). Structural brain substrates of the deficits observed on the BEARNI test in alcohol use disorder and Korsakoff’s syndrome. Journal of Neuroscience Research, 101(1), 130–142. 10.1002/jnr.25132 36200527

[IMAG.a.1205-b45] Rüb, U., Stratmann, K., Heinsen, H., Del Turco, D., Ghebremedhin, E., Seidel, K., den Dunnen, W., & Korf, H.-W. (2016). Hierarchical distribution of the tau cytoskeletal pathology in the thalamus of Alzheimer’s disease patients. Journal of Alzheimer’s Disease, 49(4), 905–915. 10.3233/JAD-150639 26519431

[IMAG.a.1205-b46] Scoville, W. B., & Milner, B. (1957). Loss of recent memory after bilateral hippocampal lesions. Journal of Neurology, Neurosurgery, and Psychiatry, 20(1), 11–21. 10.1136/jnnp.20.1.11 13406589 PMC497229

[IMAG.a.1205-b47] Segobin, S., Ambler, M., Laniepce, A., Platel, H., Chételat, G., Groussard, M., & Pitel, A.-L. (2023). Korsakoff’s syndrome and Alzheimer’s disease—Commonalities and specificities of volumetric brain alterations within Papez Circuit. Journal of Clinical Medicine, 12(9), Article 9. 10.3390/jcm12093147 PMC10179200 37176588

[IMAG.a.1205-b48] Segobin, S., Haast, R. A. M., Kumar, V. J., Lella, A., Alkemade, A., Bach Cuadra, M., Barbeau, E. J., Felician, O., Pergola, G., Pitel, A.-L., Saranathan, M., Tourdias, T., & Hornberger, M. (2024). A roadmap towards standardized neuroimaging approaches for human thalamic nuclei. Nature Reviews Neuroscience, 25(12), 792–808. 10.1038/s41583-024-00867-1 39420114

[IMAG.a.1205-b49] Segobin, S., Laniepce, A., Ritz, L., Lannuzel, C., Boudehent, C., Cabé, N., Urso, L., Vabret, F., Eustache, F., Beaunieux, H., & Pitel, A.-L. (2019). Dissociating thalamic alterations in alcohol use disorder defines specificity of Korsakoff’s syndrome. Brain: A Journal of Neurology, 142(5), 1458–1470. 10.1093/brain/awz056 30879030

[IMAG.a.1205-b50] Segobin, S., Ritz, L., Lannuzel, C., Boudehent, C., Vabret, F., Eustache, F., Beaunieux, H., & Pitel, A.-L. (2015). Integrity of white matter microstructure in alcoholics with and without Korsakoff’s syndrome. Human Brain Mapping, 36(7), 2795–2808. 10.1002/hbm.22808 25873017 PMC6869167

[IMAG.a.1205-b51] Shimamura, A. P., Jernigan, T. L., & Squire, L. R. (1988). Korsakoff’s syndrome: Radiological (CT) findings and neuropsychological correlates. Journal of Neuroscience, 8(11), 4400–4410. 10.1523/JNEUROSCI.08-11-04400.1988 3183726 PMC6569489

[IMAG.a.1205-b52] Squire, L. R., Amaral, D. G., & Press, G. A. (1990). Magnetic resonance imaging of the hippocampal formation and mammillary nuclei distinguish medial temporal lobe and diencephalic amnesia. The Journal of Neuroscience: The Official Journal of the Society for Neuroscience, 10(9), 3106–3117. 10.1523/JNEUROSCI.10-09-03106.1990 2118948 PMC6570256

[IMAG.a.1205-b53] Sullivan, E. V., & Marsh, L. (2003). Hippocampal volume deficits in alcoholic Korsakoff’s syndrome. Neurology, 61(12), 1716–1719. 10.1212/01.WNL.0000098940.31882.BB 14694035

[IMAG.a.1205-b54] Sun, X., Eastman, G., Shi, Y., Saibaba, S., Oliveira, A. K., Lukens, J. R., Norambuena, A., Thompson, J. A., Purdy, M. D., Dryden, K., Pardo, E., Mandell, J. W., & Bloom, G. S. (2024). Structural and functional damage to neuronal nuclei caused by extracellular tau oligomers. Alzheimer’s & Dementia, 20(3), 1656–1670. 10.1002/alz.13535 PMC10947977 38069673

[IMAG.a.1205-b55] Van der Linden, M., Coyette, F., Poitrenaud, J., Kalafat, M., Calicis, F., Wyns, C., & Adam, S. (2004). L’épreuve de rappel libre / rappel indice à 16 items (RL/RI-16). In M. Van der Linden, S. Adam, A. Agniel, & C. Baisset Mouly et les membres du GREMEM (Eds.), L’évaluation des troubles de la mémoire: Présentation de quatre tests de mémoire épisodique (avec leur étalonnage) (pp. 25–47). Solal Editeur.

[IMAG.a.1205-b56] Vann, S. D., & Albasser, M. M. (2009). Hippocampal, retrosplenial, and prefrontal hypoactivity in a model of diencephalic amnesia: Evidence towards an interdependent subcortical-cortical memory network. Hippocampus, 19(11), 1090–1102. 10.1002/hipo.20574 19280662

[IMAG.a.1205-b57] Vidal, J. P., Danet, L., Péran, P., Pariente, J., Bach Cuadra, M., Zahr, N. M., Barbeau, E. J., & Saranathan, M. (2024). Robust thalamic nuclei segmentation from T1-weighted MRI using polynomial intensity transformation. Brain Structure & Function, 229(5), 1087–1101. 10.1007/s00429-024-02777-5 38546872 PMC11147736

[IMAG.a.1205-b58] Visser, P. J., Krabbendam, L., Verhey, F. R. J., Hofman, P. a. M., Verhoeven, W. M. A., Tuinier, S., Wester, A., Berg, Y. W. M. M. V. D., Goessens, L. F. M., Werf, Y. D. V. D., & Jolles, J. (1999). Brain correlates of memory dysfunction in alcoholic Korsakoff’s syndrome. Journal of Neurology, Neurosurgery & Psychiatry, 67(6), 774–778. 10.1136/jnnp.67.6.774 10567496 PMC1736682

[IMAG.a.1205-b59] Williams, B., Nguyen, D., Vidal, J. P., & Saranathan, M. (2024). Thalamic nuclei segmentation from T1-weighted MRI: Unifying and benchmarking state-of-the-art methods. Imaging Neuroscience, 2, 1–16. 10.1162/imag_a_00166 PMC11873765 40041300

[IMAG.a.1205-b60] Wilson, S., Bair, J. L., Thomas, K. M., & Iacono, W. G. (2017). Problematic alcohol use and reduced hippocampal volume: A meta-analytic review. Psychological Medicine, 47(13), 2288–2301. 10.1017/S0033291717000721 28374654 PMC5595646

[IMAG.a.1205-b61] Yoneoka, Y., Seki, Y., & Akiyama, K. (2021). ‘Vascular’ Korsakoff syndrome with bilaterally damaged mammillothalamic tracts: Insights into the pathogenesis of ‘acute’ Korsakoff syndrome as acute-onset irreversible anterograde amnesia. Cureus, 13(11), e19472. 10.7759/cureus.19472 34912613 PMC8664363

[IMAG.a.1205-b62] Yushkevich, P. A., Piven, J., Cody Hazlett, H., Gimpel Smith, R., Ho, S., Gee, J. C., & Gerig, G. (2006). User-guided 3D active contour segmentation of anatomical structures: Significantly improved efficiency and reliability. NeuroImage, 31(3), 1116–1128. 10.1016/j.neuroimage.2006.01.015 16545965

[IMAG.a.1205-b63] Yushkevich, P. A., Pluta, J. B., Wang, H., Xie, L., Ding, S.-L., Gertje, E. C., Mancuso, L., Kliot, D., Das, S. R., & Wolk, D. A. (2015). Automated volumetry and regional thickness analysis of hippocampal subfields and medial temporal cortical structures in mild cognitive impairment. Human Brain Mapping, 36(1), 258–287. 10.1002/hbm.22627 25181316 PMC4313574

[IMAG.a.1205-b64] Zahr, N. M., Pohl, K. M., Saranathan, M., Sullivan, E. V., & Pfefferbaum, A. (2019). Hippocampal subfield CA2 + 3 exhibits accelerated aging in Alcohol Use Disorder: A preliminary study. NeuroImage. Clinical, 22, 101764. 10.1016/j.nicl.2019.101764 30904825 PMC6434095

