# Supplementary material for: Dissecting medial temporal lobe from diencephalic sub-volumes: The amnesia dichotomy revisited
Source: Imaging Neurosci (Camb). 2026 Apr 13;4:IMAG.a.1205. doi: 10.1162/IMAG.a.1205 (PMC13081738; doi:10.1162/IMAG.a.1205)
Supplement: Supplementary Material [file IMAG.a.1205_supp.pdf]

Table of contents

Table of contents 1

Supplementary Method. Determination of Amyloid status. 2

Supplementary Table 1. Performance indices for all considered models compared to the selected model.. 3

Supplementary Table 2. Descriptive table of mean volume (mm^3) by group and by region. 4

Supplementary Figure 1. Volume loss by group and by region. 5

References. 6

Supplementary Method. Determination of Amyloid status.

18F-AV45 PET (Fluorine-18 florbetapir positron emission tomography) images were co-registered onto their corresponding MRI and normalized to the MNI template using deformation parameters from the T1-weighted normalization procedure. Images were then quantitatively normalized using the cerebellar grey matter as the reference region, resulting in standardized uptake value ratios (SUVRs). Amyloid uptake was extracted and averaged across AD-sensitive regions, including the frontal, temporal and parietal cortices, the precuneus, the anterior striatum, and the insular cortex (La Joie et al., 2013). SUVRs were transformed to Centiloid values by applying the standardized procedure proposed by Klunk and collaborators (Klunk et al., 2015).

For validation steps, we first assessed the zero-to-100 Centiloid scale from the same data as them (PiB-PET data from GAAIN) and confirmed that our results matched theirs, demonstrating our ability to reproduce their findings. We also preprocessed their data with our pipeline to assess any variations in results. Our in-home preprocessing yielded similar outcomes, meeting validation criteria. Given that Klunk and collaborators' procedure was designed for the PiB tracer, while our study employed Florbetapir, we applied the conversion procedure proposed by Navitsky and collaborators (2018) using their data, which encompassed both PiB- and Florbetapir-PET imaging in the same individuals. We derived the zero-to-100 Centiloid scale in PiB-PET data and then applied the conversion formula to translate Florbetapir SUVRs from PET volumes of interest into the Centiloid scale. The correlation between PiB- and Florbetapir-derived Centiloid values satisfied validation criteria. Using this conversion procedure, we processed our Florbetapir-PET scans and used a cut-off of 12 Centiloid, based on previous work (La Joie et al., 2019), to define a positive amyloid status.

Supplementary Table 1. Performance indices for all considered models compared to the selected model.

Models	R2 (cond)	R2 (marg)	ICC	RMSE	Sigma	w.AIC	w.AICc	w.BIC	Perf. Score
**Selected model**	0.428	0.213	0.274	0.779	0.808	0.279	0.279	0.082	78.94%
**Selected model with linear effect of age**	0.428	0.212	0.274	0.779	0.808	0.258	0.258	0.076	76.89%
**Selected model without Sex**	0.427	0.204	0.280	0.779	0.808	0.088	0.092	0.416	71.64%
**Selected model without Sex and with linear effect of age**	0.427	0.204	0.280	0.779	0.808	0.081	0.084	0.380	69.92%
**Selected model with education**	0.429	0.214	0.274	0.779	0.808	0.149	0.143	0.003	64.99%
**Selected model with region as random effect**	0.457	0.202	0.320	0.779	0.808	0.000	0.000	0.000	60.66%
**Selected model without sex but with education**	0.428	0.207	0.279	0.779	0.808	0.067	0.067	0.020	58.09%
**Selected model without age but with sex**	0.428	0.205	0.280	0.779	0.808	0.048	0.048	0.014	56.20%

Models were evaluated using the "*compare_performance()*" function from the performance package in R. Metrics include conditional and marginal R^2 (R^2_cond, R^2_marg), intraclass correlation coefficient (ICC), root mean square error (RMSE), residual standard deviation (sigma), model weights based on AIC, AICc, and BIC (w.AIC, w.AICc, w.BIC), and an overall performance score (Perf. Score). The selected model includes fixed effects for the interaction between GROUP and region, AGE2 (AGE modeled as quadratic: AGE + AGE2), SEX, and LATERALITY, and random intercepts for SUBJECT. Model formula in R syntax is "lmer(VOLUME ~ GROUP*REGION + AGE2 + SEX + LATERALITY + (1|SUBJECT), data)".

Supplementary Table 2. Descriptive table of mean volume (mm³) by group and by region.

	AUD	KS	aMCI	dAD	HC
**Medial Temporal Lobe**					
Anterior hippocampus	1632 (242) [1021;2498]	1427 (268) [984;2010]	1510 (294) [932;2048]	1456 (201) [1062;1796]	1727 (285) [1154;2514]
Posterior hippocampus	1590 (190) [1069;2061]	1439 (151) [1170;1815]	1449 (238) [931;2062]	1436 (202) [1094;1871]	1677 (213) [1227;2179]
Entorhinal cortex	517 (77) [360;776]	453 (83) [302;643]	448 (92) [262;670]	418 (84) [264;603]	528 (83) [355;741]
Perirhinal cortex	2265 (305) [1543;3028]	1951 (324) [1341;2767]	2067 (380) [1370;2898]	2021 (377) [1255;3054]	2374 (359) [1659;3494]
Parahippocampal cortex	909 (142) [614;1260]	839 (100) [670;1044]	922 (145) [615;1299]	900 (128) [647;1174]	1023 (161) [728;1547]
**Thalamus**					
Anterior thalamic nuclei	94 (26) [23;159]	59 (22) [12;121]	77 (20) [18;122]	75 (20) [30;106]	115 (34) [35;212]
Mediodorsal thalamic nuclei	568 (70) [389;794]	424 (82) [274;596]	554 (58) [444;686]	531 (68) [319;659]	650 (94) [453;935]
Mammillothalamic tract	37 (7) [24;59]	24 (7) [13;37]	36 (6) [24;51]	36 (6) [23;51]	42 (8) [26;63]

Means are specified for each group and each region (sd) [range]
Abbreviations: AUD=patients with alcohol use disorders; KS= patients with Korsakoff's Syndrome; aMCI=patients with amnestic-type mild cognitive impairment; dAD= patients with Alzheimer's Disease at dementia stage; HC = healthy control participants.

Supplementary Figure 1. Volume loss by group and by region.

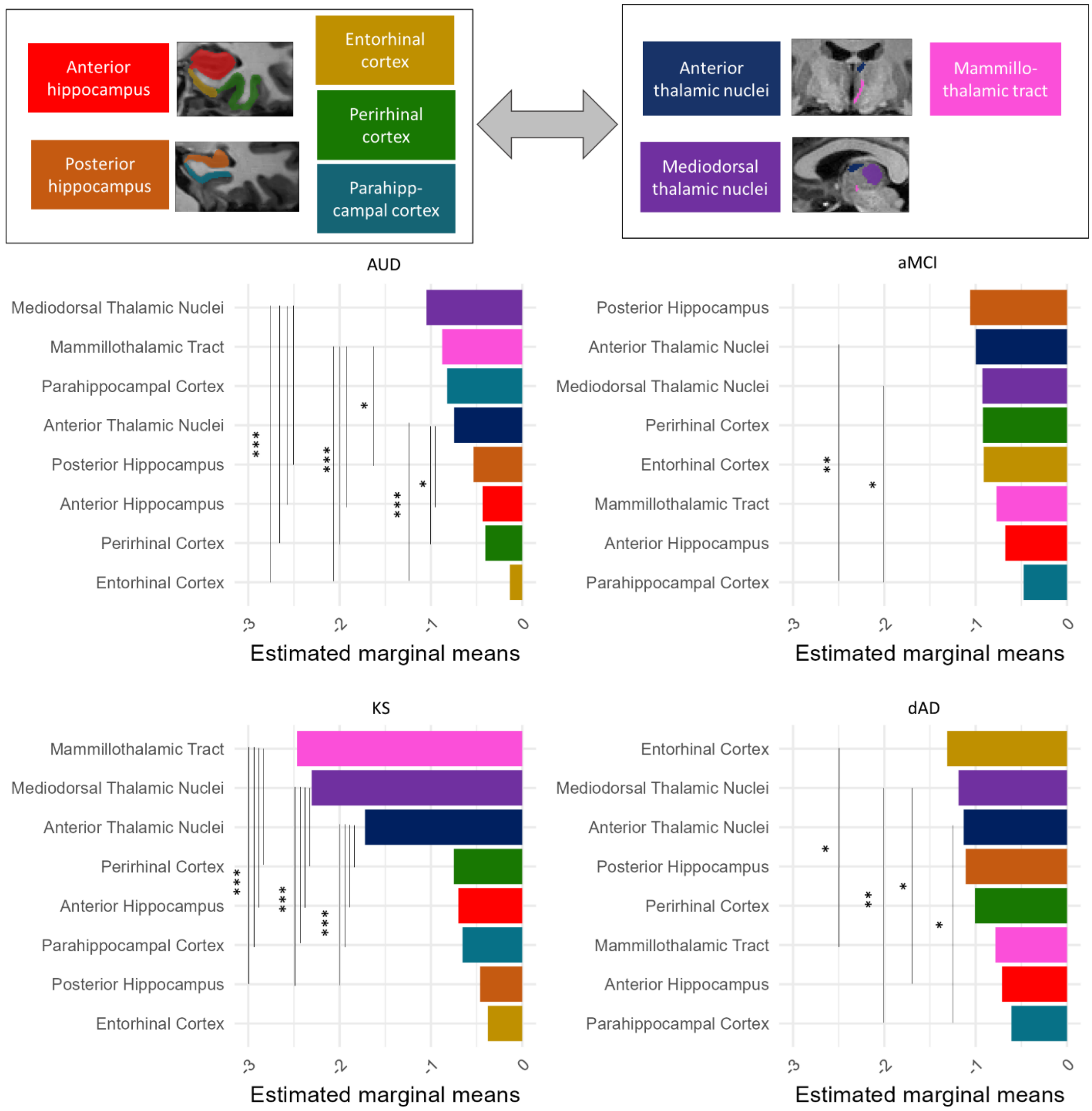

Estimated marginal means derived from the mixed linear models are represented for each group and each region (color code represents each investigated region, as represented in the top of the figure). Pairwise-comparisons were planned to compare subregions between different structures only (i.e., comparing all MTL subregions to all thalamic nuclei, but comparisons within and among MTL subregions or within and among thalamic nuclei were not performed). Statistical differences are flagged (*** p<0.001; ** p<0.01; *p<0.05).

References.

Klunk, W. E., Koeppe, R. A., Price, J. C., Benzinger, T. L., Devous, M. D., Jagust, W. J., Johnson, K. A., Mathis, C. A., Minhas, D., Pontecorvo, M. J., Rowe, C. C., Skovronsky, D. M., & Mintun, M. A. (2015). The Centiloid Project: Standardizing quantitative amyloid plaque estimation by PET. *Alzheimer's & Dementia : The Journal of the Alzheimer's Association*, *11*(1), 1—15.e1—4. https://doi.org/10.1016/j.jalz.2014.07.003

La Joie, R., Ayakta, N., Seeley, W. W., Borys, E., Boxer, A. L., DeCarli, C., Doré, V., Grinberg, L. T., Huang, E., Hwang, J.-H., Ikonomovic, M. D., Jack, C., Jagust, W. J., Jin, L.-W., Klunk, W. E., Kofler, J., Lesman-Segev, O. H., Lockhart, S. N., Lowe, V. J., … Rabinovici, G. D. (2019). Multisite study of the relationships between *antemortem* [11C]PIB-PET Centiloid values and *postmortem* measures of Alzheimer's disease neuropathology. *Alzheimer's & Dementia*, *15*(2), 205–216. https://doi.org/10.1016/j.jalz.2018.09.001

La Joie, R., Perrotin, A., de La Sayette, V., Egret, S., Doeuvre, L., Belliard, S., Eustache, F., Desgranges, B., & Chételat, G. (2013). Hippocampal subfield volumetry in mild cognitive impairment, Alzheimer's disease and semantic dementia. *NeuroImage: Clinical*, *3*, 155–162. https://doi.org/10.1016/j.nicl.2013.08.007

Navitsky, M., Joshi, A. D., Kennedy, I., Klunk, W. E., Rowe, C. C., Wong, D. F., Pontecorvo, M. J., Mintun, M. A., & Devous Sr., M. D. (2018). Standardization of amyloid quantitation with florbetapir standardized uptake value ratios to the Centiloid scale. *Alzheimer's & Dementia*, *14*(12), 1565–1571. https://doi.org/10.1016/j.jalz.2018.06.1353
